# Influences of Endplate Removal and Bone Mineral Density on the Biomechanical Properties of Lumbar Spine

**DOI:** 10.1371/journal.pone.0076843

**Published:** 2013-11-07

**Authors:** Yang Hou, Wen Yuan, Jian Kang, Yang Liu

**Affiliations:** 1 Department of Orthopaedic Surgery, Changzheng Hospital, Second Military Medical University, Shanghai, China; 2 Department of Orthopaedic Surgery, the Eighty-Eighth Military Hospital, Tai An, China; Van Andel Institute, United States of America

## Abstract

**Purpose:**

To investigate (1) effects of endplate removal and bone mineral density (BMD) on biomechanical properties of lumbar vertebrae (2) whether the distributions of mechanical strength and stiffness of endplate are affected by BMD.

**Methods:**

A total of thirty-one lumbar spines (L1-L5) collected from fresh cadavers were used in this study. Bone density was measured using lateral DEXA scans and parts of samples were performed with partial or entire endplate removal. All the specimens were divided into three BMD groups. According to endplate integrity of the lumbar vertebrae, each BMD group was then divided into three subgroups: subgroup A: intact endplate; subgroup B: central region of endplate removal; subgroup C: entire endplate removal. The axial compression test was conducted with material testing system at a speed of 2mm/min. The experimental results were statistically analyzed using SPSS 17.0.

**Results:**

(1) Significant differences of biomechanical properties occurred among normal BMD, osteoporotic and serious osteoporotic group (*P*<0.05). (2) Spearman analysis showed that BMD was positively correlated with the failure load and stiffness of lumbar vertebrae. (3) For each BMD group, significant differences of biomechanical properties were found between subgroup A and C, and between subgroup B and C (*P*<0.05). (4) For each BMD group, there was no statistical difference of biomechanical properties between subgroup A and B (*P*>0.05).

**Conclusions:**

Entire endplate removal can significantly decrease the structural properties of lumbar vertebrae with little change in biomechanical properties by preservation of peripheral region of the endplate. BMD is positively correlated to the structural properties of the lumbar vertebrae.

## Introduction

During the interbody fusion, structural struts such as prosthetic devices, autografts and allografts are implanted between two adjacent vertebrae to provide structural support following removal of diseased or damaged tissue from the spinal column [1]. The implants or grafts are expected to maintain the stability of the spine. A common feature of all these interbody implants is that they rely on the vertebral bodies for support. Settling or subsidence of these struts into the vertebral body can be a significant complication resulting in deformity, compromise of neural elements, and unfavorable biology leading to nonunion [2,3]. To prevent implant subsidence, the graft–endplate interface must have sufficient strength, which is determined by bone mineral density (BMD) and contact area, to resist the large in vivo loading [1]. Therefore modifying implants to engage the stronger regions of endplates may significantly reduce subsidence incidence. We have previously reported on the regional variation in the biomechanical properties of lumbar endplates [4]. Our results showed that the peripheral area of lumbar endplate was stronger than the central area and the strongest region was the posterolateral area in front of the pedicles. There was an increasing tendency in compressive strength of lumbar endplate from L1 to L5 and BMD was positively correlated with strength of lumbar endplates.

 In addition to the mechanical strength of the endplate, the vascular ingrowth into the graft has also been considered important for successful interbody fusion without implant subsidence [5]. The biological process of neovascularization in the setting of bone graft transplantation has been well described by the Boden SD et al [6]. Some surgeons advocate the use of preparing the endplate until punctate bleeding occurs [5,7,8]. Although this may facilitate healing, the decrease in the vertebral mechanical strength leading to subsidence is a potential risk. These two factors are difficult to control concurrently, and therefore, an effort to obtain an optimal balance between these two factors should be strived for. According to the results of our previous study [4], we assume that the central region of endplate with weaker strength can be removed without significant effect on the mechanical strength of lumbar vertebrae, which has implications for graft maintenance during interbody fusion. The primary objective of this study was to test the biomechanical properties of the lumbar vertebrae with partial and complete removal of the endplate. In addition, the influence of BMD on mechanical strength and stiffness of lumbar vertebrae was also evaluated.

## Materials and Methods

### Ethics statement

This clinical study has been approved by the institutional review board of our hospital and the written informed consent has been obtained from each deceased subject's family.

### Specimen preparation

**Figure 1:**
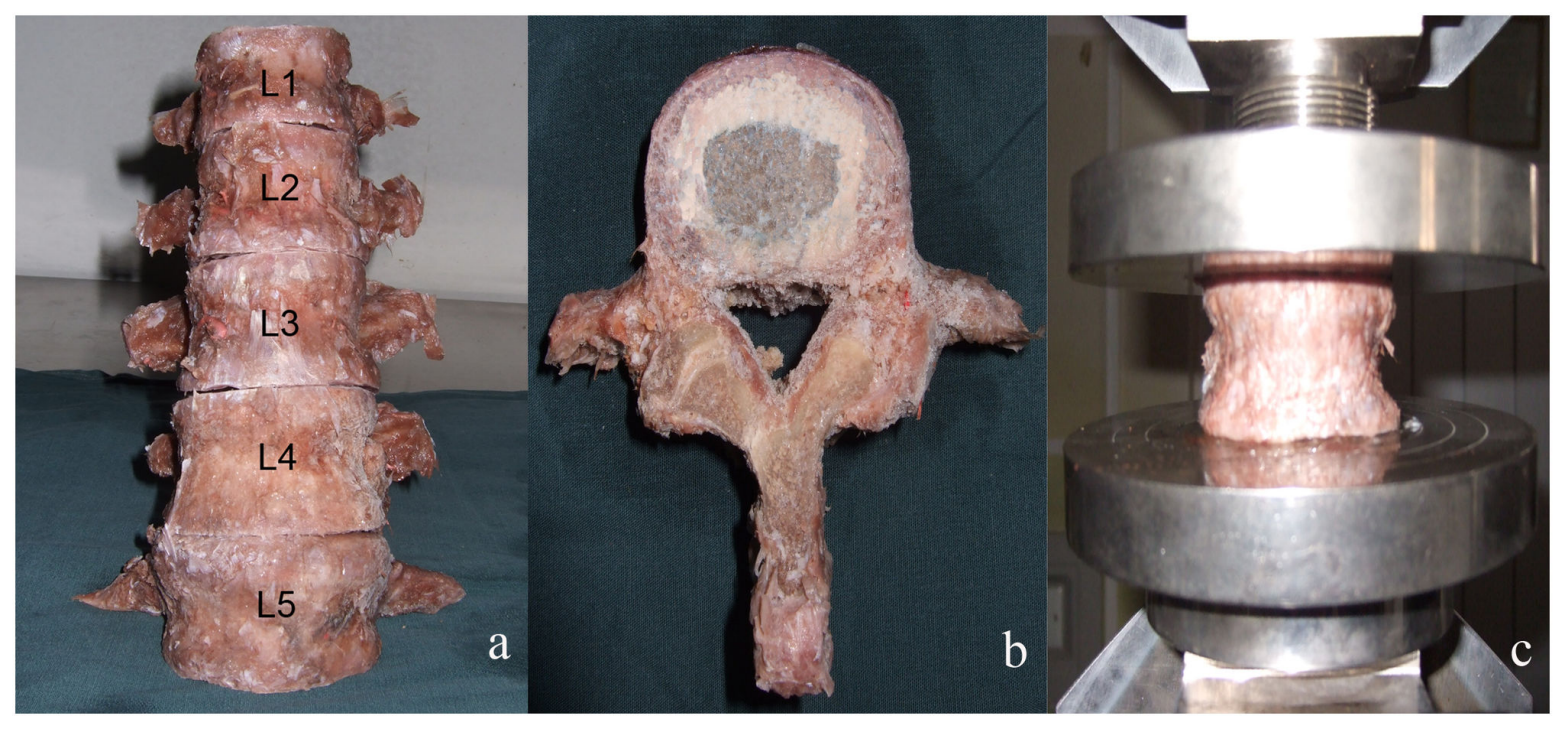
Lumbar spines (L1-L5) collected from fresh cadavers were used for biomechanical tests in the current study (a); Bony endplate was exposed by removing the soft tissue (b); After removing the posterior elements and endplate preparation, each lumbar vertebra was placed at the fixture of the material testing system and then the axial compression test was conducted under the displacement control mode (c).

A total of thirty-one lumbar spines (L1-L5, Figure 1a) were obtained from fresh cadavers (age 47–88 years, mean 67.1 years) and radiographic examination of the specimens showed no congenital deformity and tumors. All the specimens were fresh frozen and completely thawed before test. The lumbar spines were dissected through the intervertebral disc to obtain 155 isolated vertebrae (Table 1). The bony endplates were exposed using a scalpel to remove the soft tissue of the disc (Figure 1b). Lateral dual energy X-ray absorptiometry (DEXA) scans were used to record the BMD of each lumbar vertebrae. According to the diagnosis standard of osteoporosis designed by World Health Organization [9] (Table 2), the 155 vertebrae were divided into 3 groups: normal group (n=66); osteoporotic group (n=53); and serious osteoporotic group (n=36). Specimens belonging to the normal and osteopenia category were defined as the normal group. The osteoporotic group included specimens belonging to the osteoporosis category. The serious osteoporotic group consisted of specimens belonging to the severe osteoporosis category. The table 3 shows the proportion of different lumbar segments among the three BMD groups. The chi-square test was used to compare the constituent ratio of different BMD groups and no statistical difference of the constituent ratio was found among the three BMD groups (*P*>0.05). 

**Table 1:** Sample size and BMD values of three groups.

	sample size	BMD(g/cm^2^)
normal BMD group	66	1.081±0.135
osteoporotic group	53	0.784±0.143
serious osteoporotic group	36	0.386±0.091

**Table 2:** Criteria for osteoporosis.

category	BMD
normal	BMD<1 SD below young adult reference range
osteopenia	BMD 1-2.5 SD below young adult reference range
osteoporosis	BMD>2.5 SD below young adult reference mean
severe osteoporosis	BMD>2.5 SD below young adult reference mean, plus 1 or more fragility fractures

**Table 3:** The proportion of lumbar segments amongst the three BMD groups.

	sample size	L1	L2	L3	L4	L5
normal BMD group	66	12 (18.2%)	12 (18.2%)	13 (19.7%)	14 (21.2%)	15 (22.7%)
osteoporotic group	53	13 (24.5%)	12 (22.6%)	10 (18.9%)	10 (18.9%)	8 (15.1%)
serious osteoporotic group	36	6 (16.7%)	7 (19.4%)	8 (22.2%)	7 (19.4%)	8 (22.2%)

**Figure 2:**
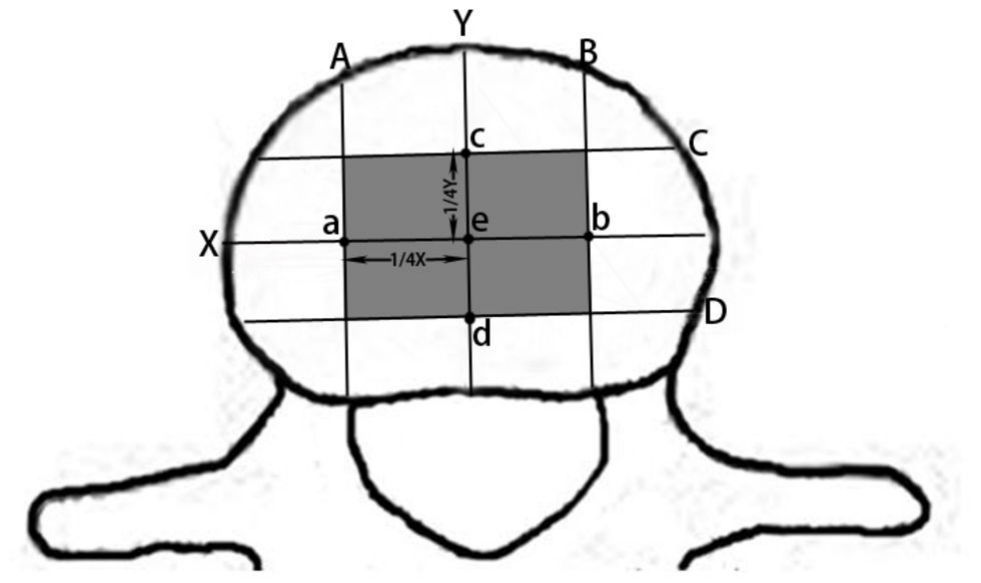
The central region of endplate removed in this study was defined as the rectangular area surrounded by two vertical lines (A, B) and two horizontal lines (C, D) which pass through the quarter-points of the x-axis and y-axis across lumbar endplate surface respectively.

 The specimens in each BMD group were divided into 3 subgroups: subgroup A: preservation of intact endplate; subgroup B: removal of central region of the superior and inferior endplate; subgroup C: entire removal of the superior and inferior endplate. To make removal of central region of different endplates have comparability, coordinate system was created directly on the surface of the lumbar endplate (Figure 2). The median sagittal diameter of the lumbar endplate was regarded as y-axis and the midpoint of y-axis was defined as origin (e). The x-axis was drawn across the origin, and the lengths of the coordinate axis were measured accurately with a vernier caliper. The quarter-points of x-axis and y-axis were determined and marked with a permanent pen. Two vertical lines (A, B) perpendicular to the x-axis were drawn across the quarter-points (a, b) of the x-axis and two horizontal lines (C, D) perpendicular to the y-axis were drawn across the quarter-points(c, d) of the y-axis. The central region removed was defined as the rectangular area surrounded by these four lines.

**Figure 3:**
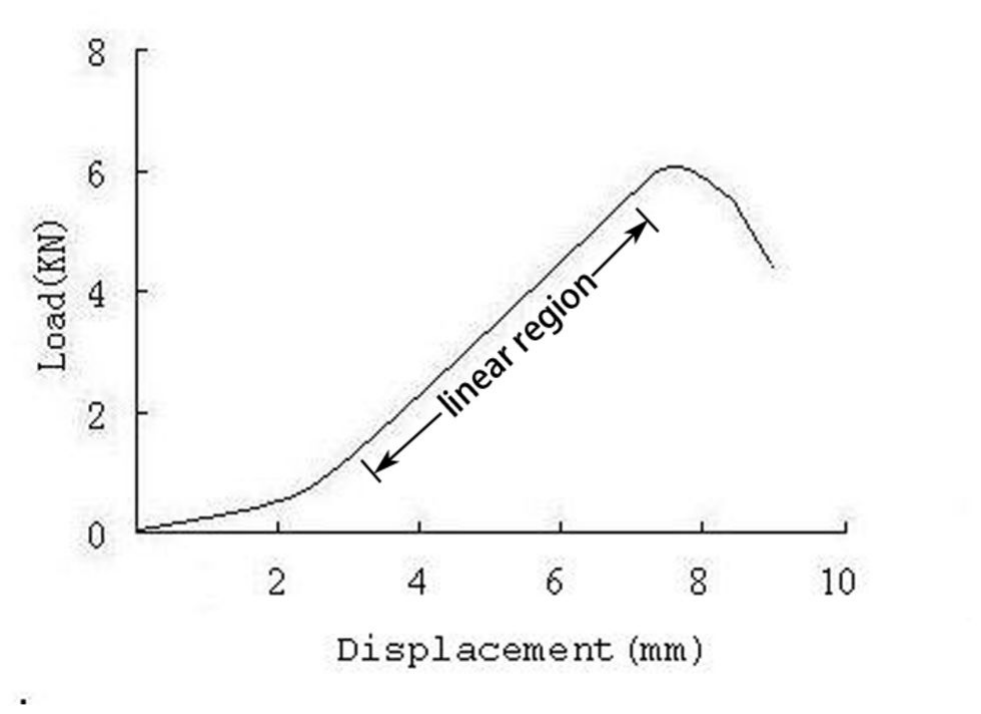
As shown in the load-displacement of the lumbar vertebrae, the compressive strength at the first significant decrease of slope of the load displacement curve was the failure load and the stiffness was the slope of linear region of load-displacement curve.

 After removing the posterior elements of each vertebra using a bone saw, the specimens were placed at the fixture of the material testing system (858 mini bionixII, Madison WI, Figure 1c). The axial force was loaded under displacement control mode. A 100N preload was applied to remove some of the slack from the mechanical testing system and then the axial compression test was conducted at a speed of 2mm/min. The load-displacement curve of each lumbar vertebrae was recorded by the computer controlled data-acquisition system. The test was stopped when the force dropped more than 50% from the maximum load. The failure load was defined as the compressive strength at the first decrease of the loading slope (Figure 3). The stiffness was the slope of the linear region of the load-displacement curve based on a linear regression analysis of the data set. 

### Data analysis

The relationship between the failure load or stiffness and BMD was analyzed by analysis of variance (ANOVA). Spearman correlation assay was also used to investigate the correlation between them and the coefficient of correlation (r_s_) indicating the linear relationship between a dependent-variable and an independent variable was determined [10]. Data of specimens in subgroup A, B, C of each BMD group were statistically analyzed by ANOVA and multiple comparisons between groups were performed using the post hoc Student-Newman-Keuls (SNK) test. The significance level of the statistical analysis was set at 0.05. The testing results were expressed in terms of mean and the standard deviation. The data were analyzed by SPSS 17.0 software.

## Results

Tabular summaries of all failure load and stiffness data were included in Table 4. For the lumbar vertebrae with intact endplate, the analysis of variance showed that differences of the failure load and stiffness in three BMD groups were statistically significant (*P*<0.05) and significant differences of the failure load and stiffness were also found by multiple comparisons among three BMD groups using SNK test (*P*<0.05). For lumbar specimens with partial or entire endplate removal, there were also significant differences of the failure load and stiffness among three BMD groups (*P*<0.05) and the SNK test also revealed significant differences of the failure load and stiffness in multiple comparisons (*P*<0.05).

**Table 4:** Measuring results of failure load and stiffness in three groups.

	normal BMD G			osteoporotic G			serious osteoporotic G		
	A	B	C	A	B	C	A	B	C
sample size	22	22	22	18	18	17	12	12	12
failure load (KN)	6.93±1.86	6.64±1.76	3.74±0.91	3.37±0.84	3.17±0.74	1.32±0.43	1.68±0.70	1.44±0.78	0.52±0.65
Stiffness (KN/mm)	2.18±0.69	2.07±0.49	1.23±0.29	1.24±0.34	1.19±0.41	0.54±0.17	0.82±0.27	0.80±0.23	0.31±0.1 9

A, B, C stand for the subgroup A with intact endplate, subgroup B with central region of endplate removal, and subgroup C with entire endplate removal respectively. The failure load and stiffness are shown in terms of mean±standard deviation.

**Figure 4:**
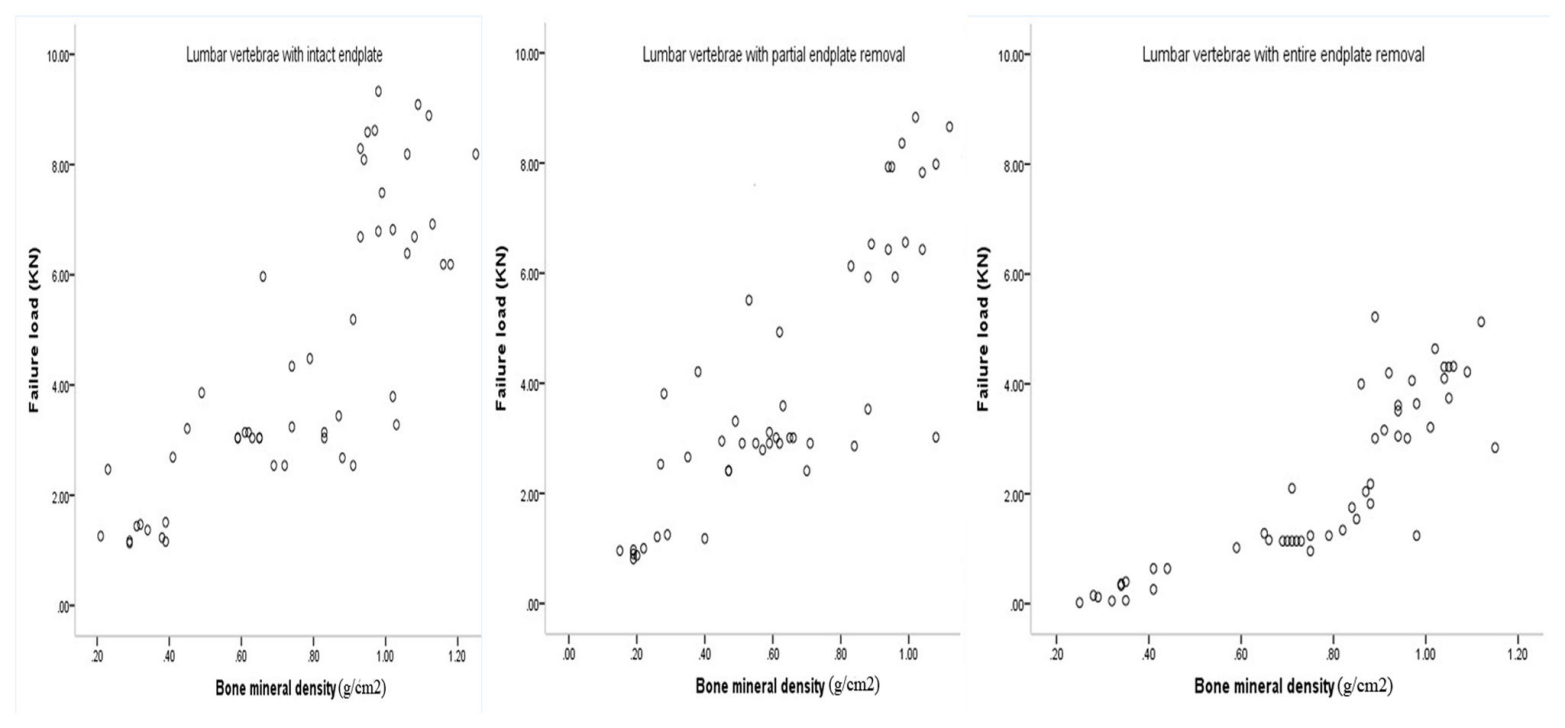
Scatter plots showing relationship between BMD and failure load in subgroup A, B and C.

**Figure 5:**
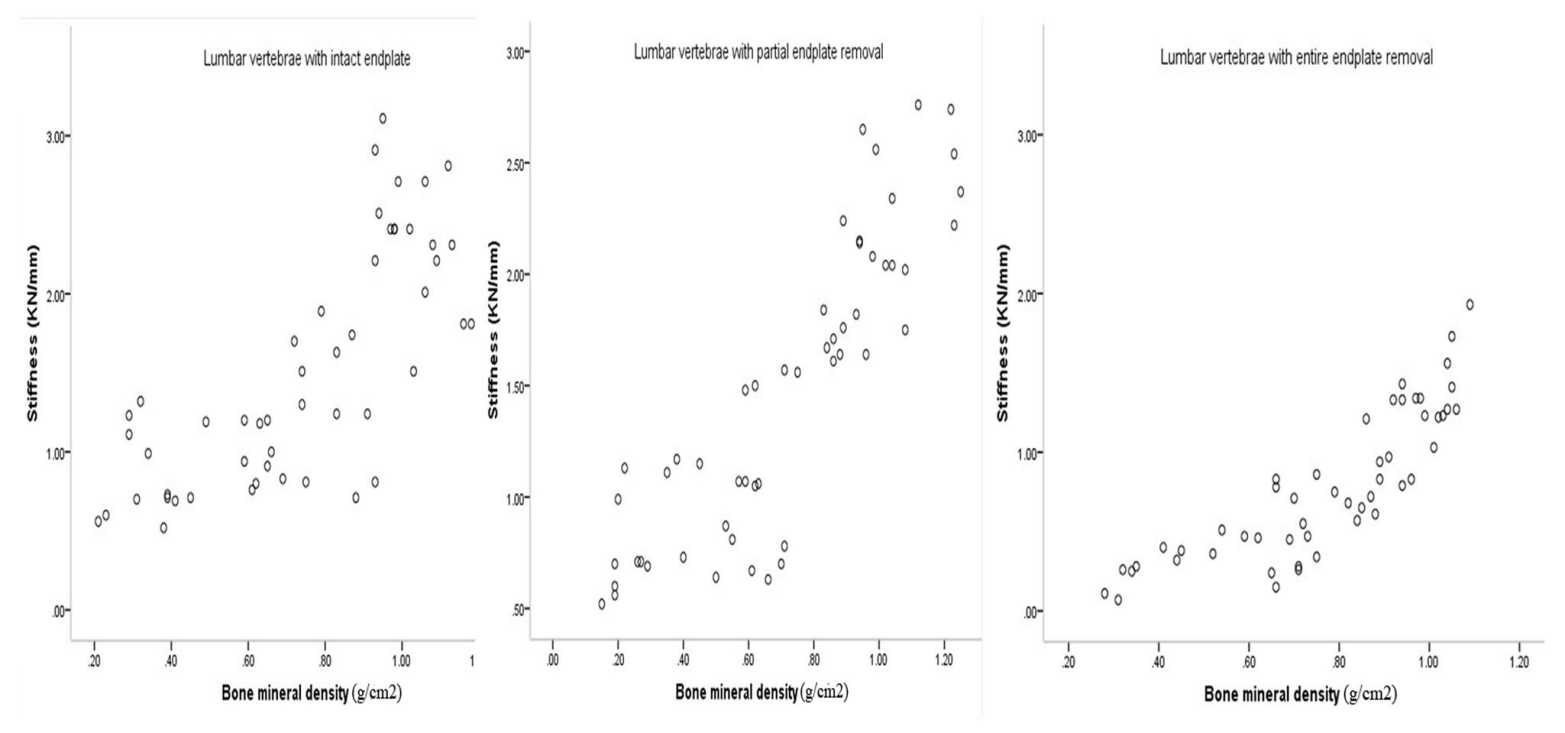
Scatter plots showing relationship between BMD and stiffness in subgroup A, B and C.

According to the Spearman correlation analysis, the failure loads of lumbar vertebrae with intact endplates were positively correlated with BMD (r_s_=0.836 *P*<0.05, Figure 4). The stiffness of these specimens, which reflect ability of the vertebrae to resist deformation under axial compression, was also positively correlated with BMD (r_s_=0.793 *P*<0.05, Figure 5). Positive correlations between BMD and failure loads in lumbar vertebrae with partial endplate removal (r_s_=0.854 *P*<0.05) and entire endplate removal (r_s_=0.905 *P*<0.05) were also observed. Stiffness was found to be positively correlated to BMD in lumbar vertebrae with partial endplate removal (r_s_=0.875 *P*<0.05) and entire endplate removal (r_s_=0.891 *P*<0.05), respectively. 

**Figure 6:**
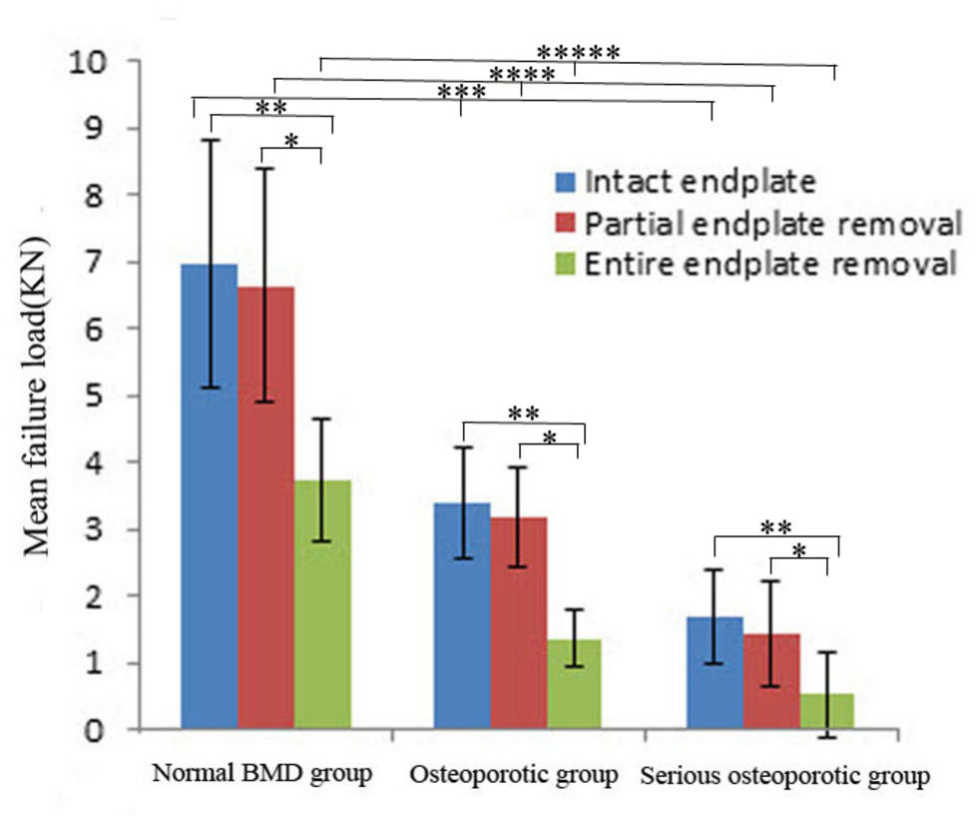
The mean failure load and standard deviation of the normal BMD, osteoporotic and serious osteoporotic group. “*” stands for the presence of statistical difference between the subgroup B and C; “**” stands for the presence of statistical difference between the subgroup A and C; “***” stands for the statistical intergroup difference of subgroup A among the three BMD groups; “****” stands for the statistical intergroup difference of subgroup B among the three BMD groups; “*****” stands for the statistical intergroup difference of subgroup C among the three BMD groups.

**Figure 7:**
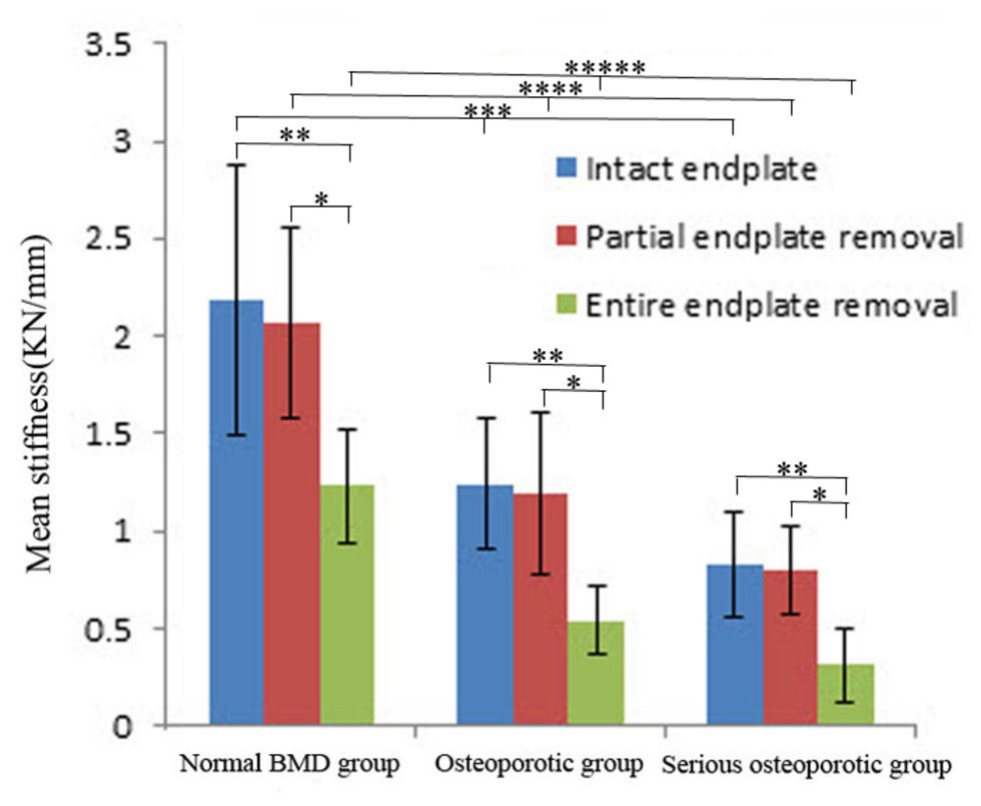
The mean stiffness and standard deviation of the normal BMD, osteoporotic and serious osteoporotic group. “*” stands for the presence of statistical difference between the subgroup B and C; “**” stands for the presence of statistical difference between the subgroup A and C; “***” stands for the statistical intergroup difference of subgroup A among the three BMD groups; “****” stands for the statistical intergroup difference of subgroup B among the three BMD groups; “*****” stands for the statistical intergroup difference of subgroup C among the three BMD groups.

 According to ANOVA and SNK results, significant differences of the failure load and stiffness were found between subgroup A and C, and between subgroup B and C (*P*<0.05, Figure 6, 7) in each BMD group. This indicated that lumbar vertebrae with complete removal of endplate were weak compared with those with preservation of the whole or peripheral region of endplate. No statistical difference of the failure load and stiffness occurred between subgroup A and B (*P*>0.05), and this showed that removal of central region of the endplate did not influence the failure load and stiffness of lumbar vertebrae. 

The mean failure load and stiffness of the lumbar vertebrae in the normal group decreased 46.1% and 43.4% when the entire endplate was removed relative to the intact endplate. With the entire endplate removal, the mean failure load and stiffness of the lumber vertebrae in the osteoporotic group decreased 60.8% and 56.6%, and the decreases of the mean failure load and stiffness were 69.0% and 62.0% in the serious osteoporotic group. The ANOVA test showed that the decreases of the failure load and stiffness were found to be statistically different among the three BMD groups(*P*<0.05). The magnitudes of decreases in failure load and stiffness were found to be significantly different between the normal group and the osteoporotic group, and between the normal group and seriours osteoporotic group by SNK test(*P*<0.05). No statistical difference was found between the osteoporotic group and the serious osteoporotic group (*P*>0.05, Table 4). With entire endplate removal, the serious osteoporotic group had the highest decrease of failure load and stiffness. The normal group had the lowest decrease and the osteoporotic group was in between.

## Discussion

Vertebral endplates are the top and bottom portions of the vertebral bodies that interface with the vertebral discs. The vertebral endplate is composed of an inner bony and outer cartilaginous endplates [11]. The vertebral endplates of adults are typically less than 1 mm thick, and although this varies considerably across the surface of any disc, there is a tendency for the endplate to be thinnest in the central region adjacent to the nucleus pulposus [12]. In our previous study, results of histologic research showed that the vertebral endplate was actually not the genuine cortical bone and it was a porous layer structure with involvement of trabeculae. The central region of the bony endplate was porous and thinner compared with the peripheral region [4]. The anatomic studies also showed that the distance between trabeculae increase in the center of the vertebra, where the basivertebral vessels run into the center of the bone [13,14]. Due to the weak strength of the cancellous bone of vertebrae, the bony endplate plays a major role in maintaining biomechanical properties of vertebrae and increasing the compressive strength of the vertebral body to prevent implants subsidence during the interbody fusion.

 It was reported that the structural properties of the failure load and stiffness vary significantly across the bony endplate surface, being greatest in the posterolateral region [1,4]. In addition, minimum graft–endplate contact areas required to prevent subsidence have been investigated [15] and different implant designs were compared to determine the optimal size and geometry of interbody support [16-18]. These studies were conducted in vertebrae with intact endplates. Some authors advocate that the endplate should be prepared to leave the interbody implant in contact with bleeding, cancellous bone [5,7,8]. This may be biologically advantageous in promoting healing, but its influence on biomechanical properties of vertebrae, which are associative with subsidence, has not been well investigated.

 Steffen et al [18] removed only about 25% of the total endplate area under some implants and did not observe compressive strength decrease. In the subsequent study [19], they tested the compressive strength of different implants with intact bony endplate or removal of the central bony endplate and found that neither implant design nor endplate preparation technique affected the ultimate compressive strength. However, they did not investigate removal of the entire endplate, and the presence of confounding variables and/or the inadequate statistical power may result in failure to detect the effect of endplate removal. Lim et al [5] conducted compression tests on cervical vertebrae using an 8-mm-diameter metal indenter with and without endplate removal and found that endplate removal did result in a reduction in local compressive strength. Lowe et al [20] performed the indentation test in the thoracic and lumbar spine using a 9.53-mm diameter indenter and they concluded that the posterolateral region of the endplate provides the greatest resistance to subsidence while the central region provides the least resistance. Disadvantages of these earlier studies include that the variation in indenter diameters can largely influence the biomechanical results and test sensitivity, and effects of endplate removal on mechanical strength of vertebrae as a whole cannot be determined by application of indenters. 

 In compression test, the shear may not be negligible unless the ends of a compression specimen are well lubricated and the shear can be reduced by increasing the height-to-diameter ratio, *h/d*, of the specimen. According to Hosford WF et al[21], the failure load of the specimen was actually higher than the true failure load of the material, which was not considered during this study. Cripton et al [22] investigated the influence of preload application methods on specimen kinematics through the spine flexibility tests. They concluded that the shear force occurred when the caudal constraint was added to reduce or eliminate the high artifact moments in the relatively unconstrained preload application method and the shear force increased with increasing preload magnitude for various loading directions including the flexion, extension, lateral bending and torsion. However, we only applied the axial loads on single vertebrae in the vertical direction and did not incorporate the caudal constraint on the preload application vector. Therefore, the influence of the shear force on the vertebral kinematics should be minimal in the current study. 

 Most interbody implants do not span the entire surface of the vertebrae. During the interbody fusion, the interbody cages are sometimes placed on either side of the vertebral body to decompress areas with the greatest neuroforaminal stenosis or in the anterior segments of the vertebral body to correct kyphosis. However, whether these implant placements biomechanically contribute to the postoperative subsidence has not been well investigated. This study aimed to address this issue and attempted to establish a baseline for future studies in this area. The results of this study showed that no statistical difference of the strength and stiffness was found between lumbar vertebrae with intact endplate and those with removal of central region of endplate. Compared with the former two groups, the strength and stiffness of the lumbar vertebrae with removal of entire endplate were significantly lower (*P*<0.05). These findings indicate that the endplate has biomechanical function of sharing loading of vertebrae and the mechanical strength of endplate mainly concentrate in its peripheral region, which is consistent with our previous finding that the central regions are thinner and weaker than the peripheral regions of lumbar endplates [4]. 

The current study demonstrates that removing the central region of the endplate does not affect the tested biomechanical parameters. The possible clinical relevance is that during the interbody fusion, the balance between the vascular ingrowth into the graft and maintenance of the biomechanical property of endplate can be addressed by removing the central region of endplate without significant influence of mechanical strength of endplate and thus can reduce graft subsidence as much as possible. However, the behavior of the implants was not directly tested in this study and the subsequent research is required to validate the efficacy of the implant placement based on these findings in the future.

 Osteoporosis is characterized by rapid and irreversible loss of trabecular bone tissue leading to increased bone fragility and implant subsidence occurs when the vertebrae become fragile. Belkoff et al [23] found that the strength and stiffness of osteoporotic vertebral bodies were significantly lower than those of vertebral bodies treated with bipedicular injections of various polymethylmethacrylate cements. Fan et al [24] conducted a series of biomechanical tests and concluded that there was a significant positive correlation between BMD and the biomechanical properties of lumbar vertebrae. Jost et al [17] conducted an in vitro biomechanical study to investigate the compressive behaviour of three different interbody cage designs in a human cadaveric model and demonstrated a positive relationship between BMD and failure loads of specimens. In addition, they also found that neither the cage design nor the presence of posterior instrumentation had a significant effect on the failure load of lumbar vertebrae. The current study also showed that the failure load and stiffness in each subgroup increased with an increase in BMD values, which was consistent with the previous studies. All these findings show that BMD is a potential confounder which can also influence the mechanical strength of the vertebral body in addition to the endplate and patients with low BMD may have higher risks of subsidence.

The limitation of this study is that we did not consider the degenerative status of the specimens which may also have influence on the biomechanical properties of lumbar vertebrae. Endplate failure occurs frequently in osteoporotic vertebral fractures and may be related to the development of high tensile strain. In this study, we did not test the tensile strains in the endplates, which may be related to the vertebral fractures. Recently, Fields et al [25] found that initial failure of the vertebrae is associated with high tensile strains in the endplates by micro-CT-based finite element analysis. In addition, subsidence may also be dependent on other biomechanical properties of endplate such as creep and viscoelasticity. The future research is required to test these biomechanical properties of endplate and whether removal of the central portion of the endplate would affect these biomechanical parameters. This biomechanical study indicates that the peripheral placement may provide structural support, however, during the interbody fusion, the load is transferred through the cephalad vertebral body through the implant to the caudal vertebral body and this study did not closely mimic that relationship. Additionally, a multitude of variables such as deformity correction and unilateral neuroforaminal stenosis may also contribute to the selection of graft placement. Therefore, it did not offer the best inference of actual interbody fusion clinical techniques. We think that the long-term prospective in vivo study is needed to provide the conclusive evidence concerning positioning of interbody grafts during spinal surgery.

In this study, no difference of failure load and stiffness was found between the normal and osteopenic vertebrae, so we included the osteopenic vertebrae within the normal group to facilitate comparison with other groups. The positive correlation between BMD and biomechanical properties of vertebrae suggests that patients with low BMD may have higher risks of subsidence after interbody fusion. For vertebrae with entire endplate removal, the magnitude of decreases of the failure load and stiffness in low BMD group is much higher than the normal group, which indicates that compared with patients with normal BMD, graft placement and maintenance of biomechanical function of endplates may be more important for patients with low BMD to prevent subsidence. However, the distribution of biomechanical properties did not change with BMD decrease and the peripheral region of endplate still undertook majority of axial loading of lumbar vertebrae, which may provide more strength to resist against subsidence postoperatively. 

## Conclusion

The fact that the lumbar vertebrae with entire endplate removal are significantly weaker than those with intact endplate suggests that the endplate plays an important part in maintaining biomechanical properties of lumbar vertebrae. In addition, removal of the central region of endplate does not significantly influence the mechanical strength of vertebrae, which indicates that the peripheral portion of endplate shares the major loading of lumbar vertebrae. The fact that BMD is positively correlated to biomechanical properties of lumbar vertebrae indicates that patients with low BMD may have higher risks of subsidence. With the decrease of BMD, the peripheral region of endplate still undertook majority of axial loading of lumbar vertebrae and may provide more strength to resist against subsidence after interbody fusion.
